# Exploring Default Mode and Information Flow on the Web

**DOI:** 10.1371/journal.pone.0060398

**Published:** 2013-04-24

**Authors:** Mizuki Oka, Takashi Ikegami

**Affiliations:** 1 Center for Knowledge Structuring, The University of Tokyo, Hongo, Tokyo, Japan; 2 Graduate School of Arts and Sciences, The University of Tokyo, Komaba, Tokyo, Japan; University of Zaragoza, Spain

## Abstract

Social networking services (e.g., Twitter, Facebook) are now major sources of World Wide Web (called “Web”) dynamics, together with Web search services (e.g., Google). These two types of Web services mutually influence each other but generate different dynamics. In this paper, we distinguish two modes of Web dynamics: the *reactive mode* and the *default mode*. It is assumed that Twitter messages (called “tweets”) and Google search queries react to significant social movements and events, but they also demonstrate signs of becoming self-activated, thereby forming a baseline Web activity. We define the former as the reactive mode and the latter as the default mode of the Web. In this paper, we investigate these reactive and default modes of the Web's dynamics using transfer entropy (TE). The amount of information transferred between a time series of 1,000 frequent keywords in Twitter and the same keywords in Google queries is investigated across an 11-month time period. Study of the information flow on Google and Twitter revealed that information is generally transferred from Twitter to Google, indicating that Twitter time series have some preceding information about Google time series. We also studied the information flow among different Twitter keywords time series by taking keywords as nodes and flow directions as edges of a network. An analysis of this network revealed that frequent keywords tend to become an information source and infrequent keywords tend to become sink for other keywords. Based on these findings, we hypothesize that frequent keywords form the Web's default mode, which becomes an information source for infrequent keywords that generally form the Web's reactive mode. We also found that the Web consists of different time resolutions with respect to TE among Twitter keywords, which will be another focal point of this paper.

## Introduction

Approximately 90% of the World Wide Web's (hereafter referred to as “Web”) data stream is said to have been created within the last few years [1], and the total amount of data is increasing every day. It is also said that Web data volumes are doubling every two years. This exceptional growth is mainly due to emerging social network services (SNSs), such as Twitter and Facebook. The advantage of SNSs became widely recognized after the Egyptian revolution of February 11, 2011 and the earthquake of March 11, 2011 in Tohoku, Japan. Facebook helped bring worldwide attention to the historical event in Egypt, and Twitter served as an efficient platform for communicating and obtaining information regarding the earthquake. This shift in the Web from search to communication over the last 10 years has been remarkable. People are using the Web to see what other people are doing and to share feelings and ideas.

The two types of Web services, represented here by Google and Twitter respectively, constitute the majority of the information being diffused and circulated on the Web. Google and Twitter have different memory structures. For example, only 126 out of the 3,479 unique trending topics (3.6%) from Twitter exist in the 4,597 unique hot keywords of Google [2]. These keywords are mostly associated with real-world events, celebrities, and movies. On average, 95% of the hot keywords per day are new in Google, while only 72% of the hot keywords are new in Twitter [2]. This feature is worth noting, since it reflects that retweets, replies, and mentions are prevalent in Twitter; however, such interaction among users is not possible with Google searches. Retweet is a unique feature in Twitter called retweeting (RT), whereby people repost their favorite messages (called “tweets”) on their timelines. Statistics show that half of the RT occurs within an hour and 75% in less than a day [2]. However, approximately 10% of the retweets occur a month later. This implicit cooperative feature of Twitter makes Twitter's time series different from Google's time series and as the data show, because of this, the same trending topics persist over a relatively longer period of time in Twitter.

In order to investigate these differences between Google and Twitter, we examined how these two services affect each other. How are their time series different from each other? Does information flow from Twitter to Google or vice versa? We conducted an intensive time series analysis to answer these questions, and to better characterize Web dynamics with respect to Google and Twitter. In this paper, we distinguish between two modes of Web dynamics: one is *reactive mode* and the other is *default mode*. There are examples in which Twitter and Google react strongly to social movements by producing *bursting* behaviors. This is what we call the Web's *reactive mode*. For example, a burst in the popularity of keywords such as “earthquake” or “nuclear plant” was observed on and after the earthquake of March 11, 2011 in Japan.

The Web also demonstrates aperiodic non-stationary temporal dynamics without necessarily showing bursting behavior, forming a baseline Web activity. We define this baseline activity as the Web's *default mode*. People's tweets may or may not be affected by other users' tweets appearing in their *timelines*, which shows the list of tweets from users they are following. Thus, although global information is not explicitly shared to cooperatively generate tweets that include the same keywords, these tweets may reflect a weak correlation by circulating within Twitter through users' timelines. This reminds us of the classic network theory called *weak ties* [3]. The potential for organizing informational structures and patterning through weak ties is nicely realized by the interactions mediated by timelines. We argue that Twitter as a weak tie generates a *default mode* in Web dynamics. The default mode can be an important Web mode, not only for supporting baseline activity but also for reducing uncertainty in information circulation on the Web, thereby regulating the consistency of information between the Web and the actual world.

For the purpose of revealing and characterizing the Web's default mode, we computed the transfer entropy (TE) between the time series of Twitter keywords and Google queries as well as the information transfer within keywords. TE is one of the information entropy measurements for estimating how the uncertainty of a time series is reduced by using either its preceding states or other time series [4]. TE cannot measure the causal effect but can provide a predictive measure, as discussed in [5], [6]. We hope that this study provides useful information for understanding other complex adaptive and autonomous systems, such as brain systems.

## Materials and Methods

### Data Collection

We collected two types of time series data; one is the time series data of a set of keywords contained in Twitter and the other is the time series data of the same set of keywords issued as Google queries. The data were crawled over an 11-month period using Google Trends [7] and Twitter API (Japanese tweets only) from July 16, 2011 to May 13, 2012, which is 302 days. The set of keywords was chosen by selecting the top 1,000 keywords that appeared in Twitter during this period. Google Trends only provides the volume of how many queries are issued on a daily basis, thus the time series data for Google is an aggregation of the popularity of a particular keyword that day. As for Twitter, data are available on a much finer timescale; thus, we prepared two types of time series, one in which popularity was aggregated per day and the other in which popularity was aggregated per hour. The one-day unit time series was used to compute TE between Google and Twitter, and the one-hour unit time series was used to compute TE within Twitter.

### Bursts

We define a bursting behavior as “a keyword's popularity that shows a sudden increase.” This was observed in the time series of Twitter keywords and Google queries. For example, the keyword “earthquake” bursts every time there is an earthquake. Since the most salient property of the time series is bursting behavior, counting the number of bursts is a first step toward characterizing the reactive or default modes of the time series.

A state-of-the-art burst detection method determines the local maximum of the *peaks* (e.g., log derivatives [8]). However, our Twitter dataset analysis revealed that the most dominant distributions among the 1,000 most-frequent keywords were those with log-normal distributions. Therefore, to more accurately detect the bursts in our dataset, we first determined the burst region, using the standard deviation (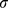) and the mean value (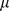) of the logarithm of popularity; second, we defined the burst region, where popularity first goes above 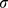 + 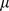 and then goes below this value; and third, we extracted the maximum popularity within the region as a peak. More precisely, 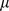 and 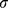 were defined as
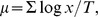


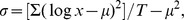
where 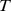 denotes the total number of points in the time series, which is determined by the time resolution (i.e., 302/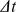). If the successive bursting time points were less than or equal to 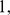 we do not consider this as a bursting period. In this paper, we take these peaks as the definition of “bursts.”

Time resolution is another complex factor to be considered. Bursts of each keyword vary with different time resolutions. For example, the keyword “good night” has 24-hour periodicity and in order to detect this periodic burst, a one-hour time resolution is adequate. If the time resolution is shorter than one hour, too many bursts are detected. In order to suppress periodic bursts in the time series, we should choose the adequate time resolution for each keyword, but there is no universal time resolution that is applicable to any keyword. We will come back to this point in subsequent sections.

The effects of tweets generated by bots also need to be considered. It is known that 51% of traffic on average Web sites is potentially generated by bots [9]. Bots tend to post a large number of tweets that include the same keyword in a very short time period. We can mitigate the effects of bots by removing bots that post an extreme number of tweets, say, in a few seconds [10]; however, obviously, it is not possible to remove all the bots. In fact, these indistinguishable bots can be considered as an essential part of the Web, which function to maintain overall activity that may also induce human actions. Overall, these various temporal scales organize a background temporal structure in Twitter's time series and, together with sudden bursting behavior and autonomous bot tweets, act to form the Web's temporal dynamics.

### Information Flow

The information transfer observed in Twitter keywords and Google queries is computed on the basis of TE, which was developed by Schreiber [4], Staniek and Lehnetz [11], [12], and Bertschinger [13]. TE is one of the information entropy measurements for estimating how the uncertainty of a time series is reduced by using either its preceding states or other time series. In this sense, TE is similar to Granger causality [14]–[16], which calculates the degree to which one time series drives another. However, TE has advantages over Granger causality, since TE can eliminate the false contribution from the common temporal pattern that is present in both time series when comparing two temporal time series. For this same reason, TE has an advantage over mutual information. On the other hand, TE cannot measure the causal effect but can provide a predictive measure, as discussed recently in [5], [6].

Suppose the TE between two different temporal time series is associated with the keywords 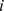 and 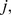 respectively. If the TE from 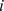 to 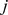 is greater than that from 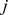 to 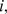 it can be said that knowing the temporal sequence of 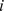 decreases the uncertainty of 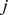 compared with the opposite case. Differing from mutual information, TE has the advantage of being able to ascertain the direction of information flow, rather than mere temporal correlation. Practically speaking, it is generally difficult to measure the causal effect without knowing the underlying equation and, since Granger causality calculates the linear approximation, it is not adequate for highly nonlinear systems, which is the case with our datasets. For these reasons, we use TE in our study.

For example, according to [4], an advantage of TE is that it can be used to evaluate the causes of epilepsy in the brain using EEG sequences [11]. Another example is comparing heartbeat and breathing sequences to evaluate which affects the other's dynamics [4]. Recently, TE has been used to successfully reveal the underlying network of information transfer in the popularity of hashtags in Twitter [17]. By using the computationally feasible quantity called permutation entropy, TE differentiates between upstream and downstream information flow in a realistic time series with a large, dynamic range of values. That is, the TE of the given pair of time series can be computed in two steps. The first step is to compute the permutation entropy of the 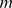 number of the possible ways of labeling local temporal patterns. The second step is to compute TE on the permutated time series.

### Transfer Entropy (TE)

Shannon entropy, with the probability distribution of 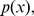 is commonly defined in the following manner (where 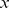 is an extracted state of a target system; e.g., time sequence 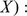


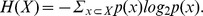
Using this notation, we define mutual information (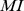) between two time series 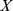 and 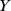 in the following manner:



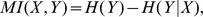
where 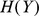 is the uncertainty of 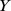 and 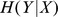 is the uncertainty of 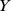 for knowing 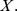 By definition, 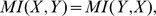 so that no causal relationship is detected with MI. By introducing the time delay, we can improve the situation, although it remains difficult to capture the direction of causality.

On the other hand, TE from 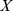 to 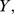 which is denoted as 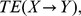 is defined by
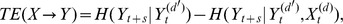
where 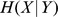 denotes the conditional entropy and 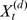 and 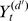 denote the past history of 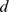 and 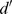 length counted from the present time (i.e., 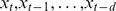 or 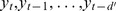), respectively. Here, we also considered the time delay effect 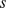 and the different time length of the past 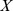 and 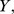 (i.e., 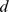 and 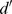), respectively. TE measures the decrease of uncertainty in the state 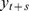 by knowing the uncertainty of 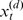 from the past history of other variables such as 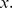 If there is no information flow from 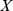 to 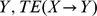 disappears but 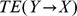 does not, as the TE is explicitly non-symmetric with respect to 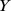 and 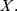


Let us express the formulas in a more explicit manner by using the probabilities of each element of the time series, such that
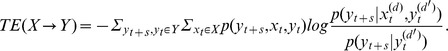
Here, 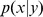 denotes conditional probability. The opposite TE is obtained in the same manner; for example,



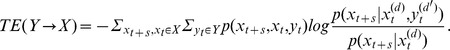
Then, we measured the direction of the information flow by comparing the TE for the pair of time series. In particular, we use the difference between 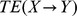 and 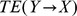 as the quantity of information flow denoted by 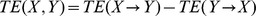 in the remainder of this paper. In order to calculate TE practically, we should discretize the continuous state flow. Because of the trends in the time series, it is difficult to discretize the state by its absolute value. Thus, we used permutation entropy as explained below to have a stable measurement.

### Permutation Entropy and Time Resolution

Bandt and Pompe [18] introduced a simple refinement of entropy with sequences that are practical and that allow feasible coding of the real-value dataset. This method is based on the re-ordering of the amplitude values of the time series 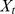  =  {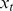} and 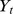  =  {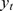}, so that the amplitudes are arranged in ascending order. Namely, 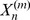  =  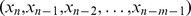 are arranged in ascending order and become 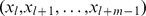 such that 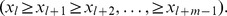 We now use the indexes of these variables instead of their amplitudes; for example, 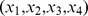 is re-ordered as 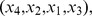 so that 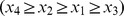 and the new temporal sequence is 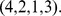 In this example, 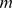 is set to 4, which generates a total of 24 patterns (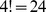). By shifting a window, we assign a number to each local time series of the length 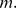 In this manner, any time series can be mapped onto a string of finite symbols, thereby allowing us to estimate the probabilities needed to compute the TE. Yet, choosing a large 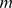 could use a large amount of computation time and resources. This is because the probabilities of the triplet 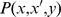 is defined on the large number of combinations; therefore, we need the same order of sampling points. In the case of both Twitter and Google, 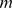 is not well estimated; thus, we had to conduct an elaborate search on the time series of both Twitter and Google by changing the dimensionality 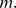


Using permutation entropy, we compute the TE of a given time series 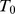 in the following manner:

1. Generate a time series 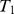 of the length 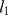 by aggregating the state 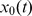 (i.e., the sum of tweets between 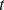 and 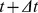) for each time steps of the original time series 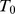 of length 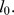 The length 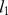 now becomes 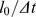 and the state now becomes 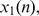 where 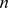 ranges from 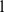 to 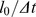 as follows,
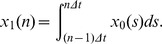

2. Redefine the time series 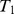 as 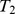 which is obtained by permuting 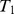 for each 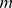 time window. By shifting the time window individually, we obtain the length 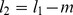 and the number of possible symbols is 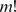.
3. Compute the TE on 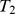 with the parameters 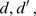 and 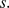 It should be remarked that 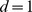 corresponds to 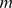 time steps in 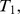 which thus correspond to 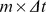 time steps in the original sequence 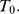

In all, we have five parameters (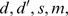 and 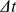) to compute the TE from the original time series. We set 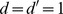 and 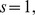 and utilize 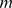 and 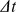 as major controlling parameters. This is based on our understanding that changing 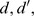 and 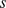 is not adequate because the values that must be chosen are also highly dependent on which 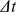 is chosen. In other words, changing 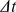 and 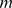 can also account for changing parameters 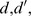 and 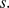 Therefore, it is preferable to fix the parameters 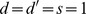 and vary the other two parameters, 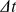 and 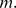 The optimal 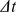 and 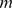 must be different for each time series; however, it is pragmatically difficult to select different 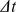 for each sequence. It may not be the best method, but we adopt our parameters empirically using the following principles: 1) For computing TE between Google and Twitter, we checked 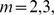 and 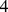 and adopted 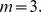 We did not use 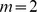 as it does not take advantage of using the re-ordering, nor 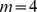 because if 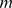 is greater than 4, it becomes too large for the number of sampling points we have for the Google time series. We changed 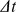 for testing how it changes the TE distribution, as will be evident in the results section. 2) For computing the TE inter-Twitter time series, we adopted 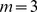 for the same reason. Since we have a relatively larger data set for the Twitter time series, we changed 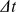 over different time resolutions from 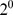 minute to 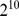 minutes to see how the change in 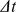 affects the results of the TE. Then, we selected one fixed value for 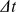 to argue the TE flow within Twitter's TE network.

## Results

The inherent patterns and dynamics of Google's and Twitter's time series were investigated to define and characterize the Web's reactive and default modes, using the top 1,000 most-frequent keywords found on Twitter. For example, the top 10 most-frequent keywords were “today,” “thing,” “people,” “RT,” “this day,” “laughter,” “now,” “best,” “I,” and “tomorrow.” In the subsequent subsections, we show the relation between the number of bursts and the frequency ranking of keywords as well as the results of TEs that quantify inherent patterns and dynamics.

### Dynamics of Frequent and Infrequent Keywords

**Figure 1:**
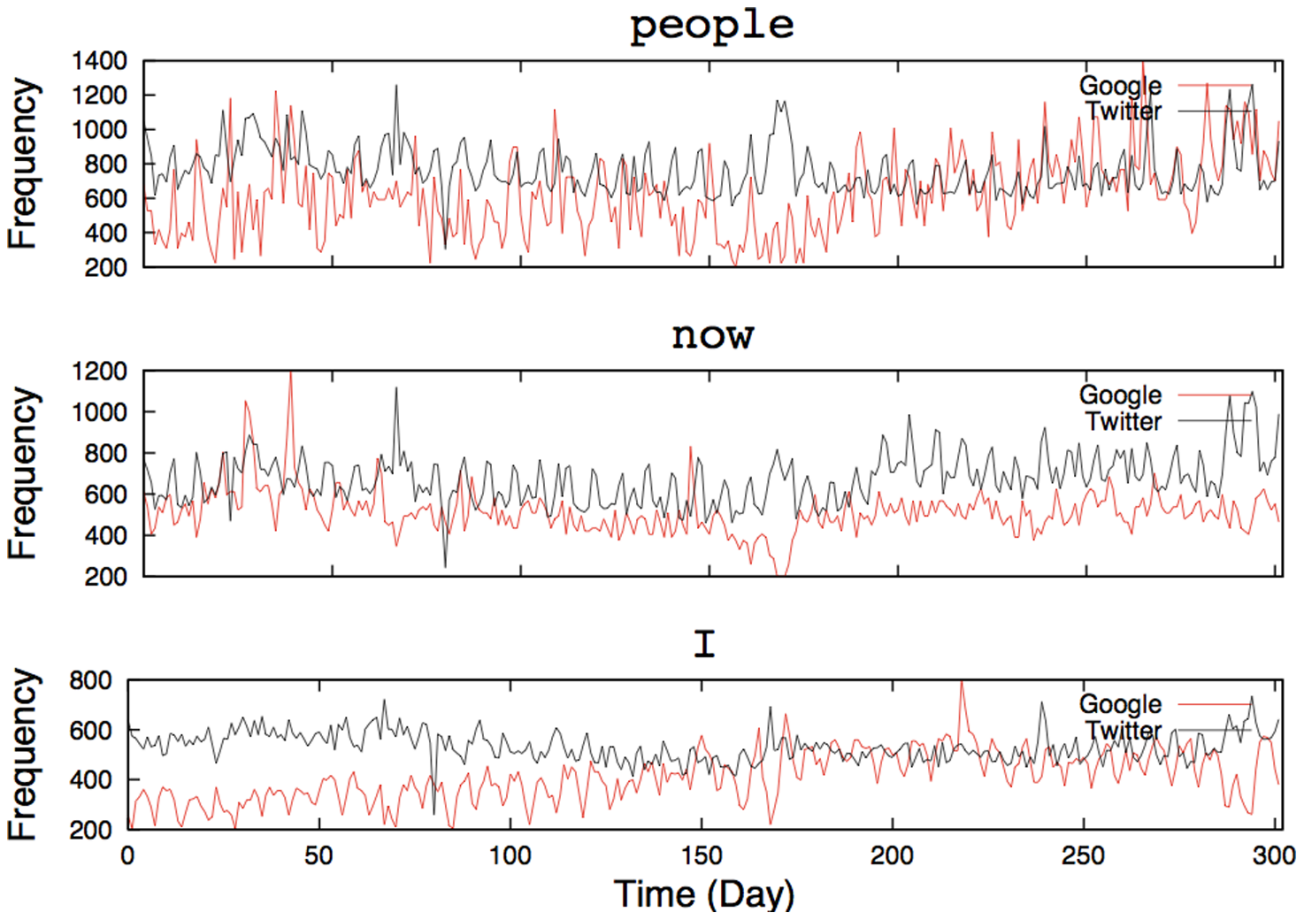
Examples of frequent keywords in Twitter keywords and Google queries. Burst behaviors are not salient and synchronization between the two time series was not observed.

**Figure 2:**
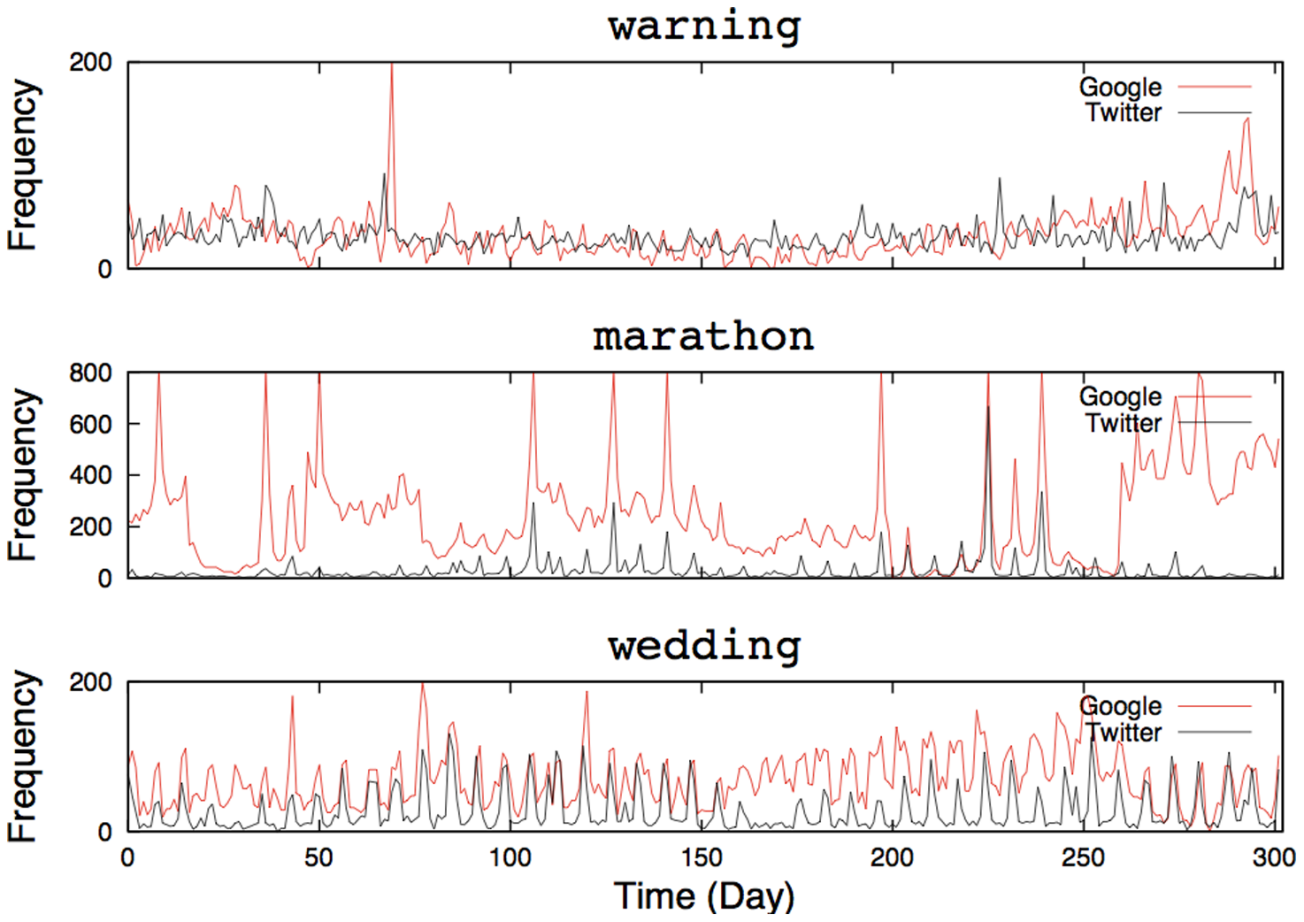
Examples of infrequent keywords in Google queries and Twitter keywords. Burst behaviors were clearly detected and synchronization between the two time series was often salient.

Figure 1 shows the temporal dynamics of some of the most-frequent keywords, such as “people” (ranked 3rd), “now” (ranked 7th), and “I” (ranked 9th). These frequent keywords form the daily life dynamics of Twitter. Interestingly, we rarely observe bursting behaviors in frequent keywords. Rather, more periodic behaviors are observed because these keywords reflect people's repetitive habits, such as tweeting more frequently during the day and less at night. This apparent periodic rhythm becomes explicit in frequently used keywords. On the other hand, Figure 2 presents examples of infrequent keywords, such as “warning” (ranked 862nd), “marathon” (ranked 930th), and “wedding” (ranked 983rd). Interestingly, these infrequent keywords show synchronization between Google's and Twitter's time series and reveal more bursting dynamics with sudden aperiodic spikes.

### Frequency of Keywords and Number of Bursts

**Figure 3:**
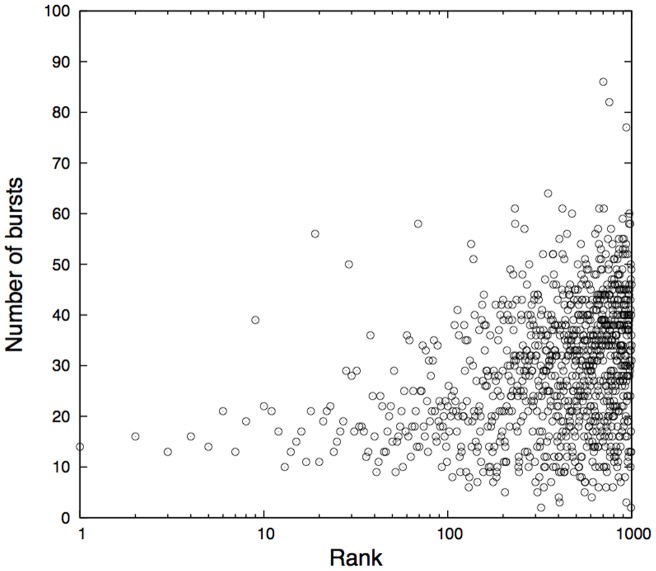
Relationship between the number of bursts and keyword frequency rankings. There was a tendency for less-frequent keywords to have a greater number of bursts and the fewer number of bursts were detected with frequent keywords.

Figure 3 depicts the relationship between the number of bursts, defined as a sudden increased popularity, and the keyword frequency ranking. As this figure shows, frequent keywords tend to have almost no bursts and infrequent keywords have more bursts. Infrequent keywords such as “warning” or “marathon,” are rarely tweeted in everyday life, but they may be triggered by real-world events that impact numerous people. We say that these infrequent keywords constitute the Web's reactive mode toward real-world events. On the other hand, frequently used keywords constitute the Web's baseline acntegrated TE of a given keyword to/from the other tivity without being activated externally. Namely, these keywords rarely respond to particular events, but continue to fluctuate by themselves. The reactive mode is sustained by external causes and the Web's baseline activity is maintained intrinsically. We call this baseline activity the default mode.

In the following sections, we show that transfer information can characterize the time series of frequent or infrequent keywords and discuss the Web's reactive and default modes in greater detail.

### Determining the Time Resolution Δ*t*

**Figure 4:**
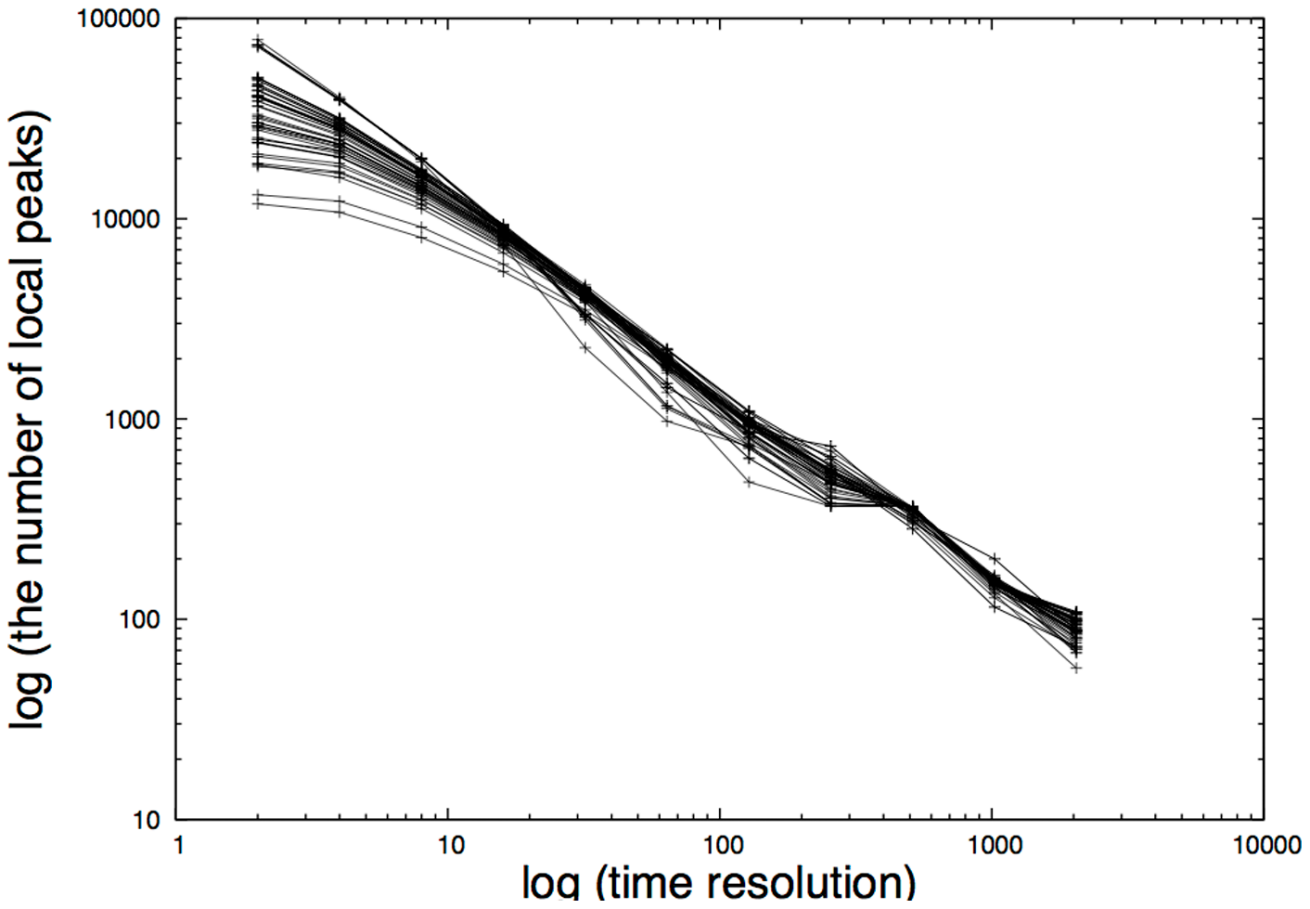
Relationship between the number of local peaks and time resolution Δ*t* in log scales. Data computed from 46 keywords are superimposed in the figure, showing that the number of local peaks obeys a power-law of up to around 2^9^(= 512) minutes.

We are free to choose a base duration for each time series 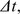 thus, we investigated how the change in 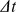 differs in the resulting time series. More specifically, we aggregated tweets from 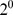 minute to 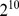 minutes and simply counted the number of local peaks (i.e., 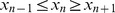). The analysis was conducted on a set of 46 keywords that were randomly chosen from 1,000 keywords. The results are presented in Figure 4. It is evident from the figure that the number of peaks approximately obeys the power-law with an exponent of around 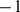 for 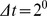 to 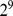 irrespective of keywords. After 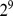 minutes, the individual difference in keywords gradually became apparent. It is evident that these two features correspond to the micro and macro time scales of Twitter, respectively. In the micro time scale in the order of minutes to an hour, it reflects each user's online tweeting action. In the macro time scale in the order of hours to a day, it reflects the mood of a society, that is, what people care about and what people are anticipating.

In computing the entropy transfer between Google and Twitter, we examine the macro time scale domain because Google only supplied a one day dataset and we are only concerned with how information is circulated at a societal level. On the other hand, in computing the entropy transfer of inter-Twitter time series, we examine the micro time scale because we are concerned with the action pattern of users. Namely, each Twitter user is basically concerned about what appears on his/her timelines and that should be part of the micro time scale.

### Information Transfer between Twitter and Google

In order to investigate the Web's reactive and default modes, we computed the TE between Twitter and Google for each keyword. As discussed above, we used the time resolution of one day as a base unit and the window size was set to 302 steps (which corresponds to 302 days) in order to satisfy the required sampling points (i.e., 216 points for 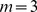). We increased the time resolution 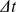 from one day to four days to see how it affects the entropy transfer.

**Figure 5:**
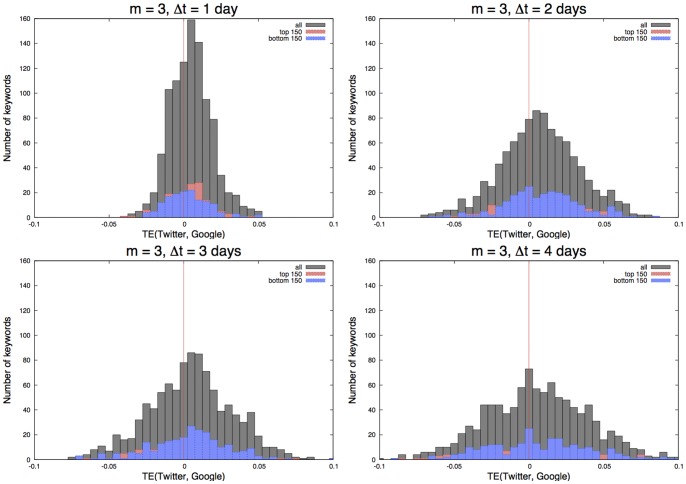
The 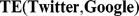 histogram for all the 1,000 keywords, and the top and bottom 150 keywords, by changing 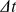 from one to four days. It was found that the information has the tendency to flow from Twitter to Google. For example, 692 keywords show that information is transferred from Twitter to Google (where 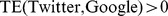) and 308 keywords show that the information is transferred from Google to Twitter (where 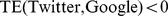) in the case of 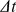 equals to one day.

Figure 5 shows the 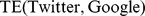 for 1,000 keywords for 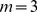 and 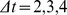 days with their plotted distribution, respectively. Further, the distribution of the top 150 and the bottom 150 most-frequent keywords is superimposed. If the TE is positive, it implies that the information transfer is from Twitter to Google, otherwise it is from Google to Twitter. It is suggested from this figure that information is transferred more from Twitter to Google than from Google to Twitter as a whole. Indeed, when examining the TE for individual keywords when 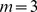 and 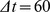 minutes, the total of 692 keywords (out of 1,000) shows that information is transferred from Twitter to Google and 308 that it is transferred from Google to Twitter.

When comparing the top 150 frequent and bottom 150 keywords, we see a weak trend indicating that the top 150 keywords transfer from Twitter to Google more than the bottom 150 do. This suggests that the Web's default mode (or frequent keywords with rare bursting behaviors) contributes to reducing uncertainty in the information circulation on the Web more than the Web's reactive mode (or infrequent keywords with many bursts).

Next, we measured the information transfer among Twitter keywords and discovered an inherent network behind the keyword network connected through TE. Based on their information transfer, we call this network among keywords the transfer entropy network (TE network); we examine this in the next section.

### Information Transfer on Twitter

Before computing the TEs of all the pairs of 1,000 keywords in Twitter, we used the same 46 keywords as those in the section entitled **Determining the Time Resolution** 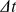 and computed the TEs of all the pairs in this set of keywords by changing the time resolution 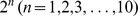 minutes to see how they affect the TEs.

**Figure 6:**
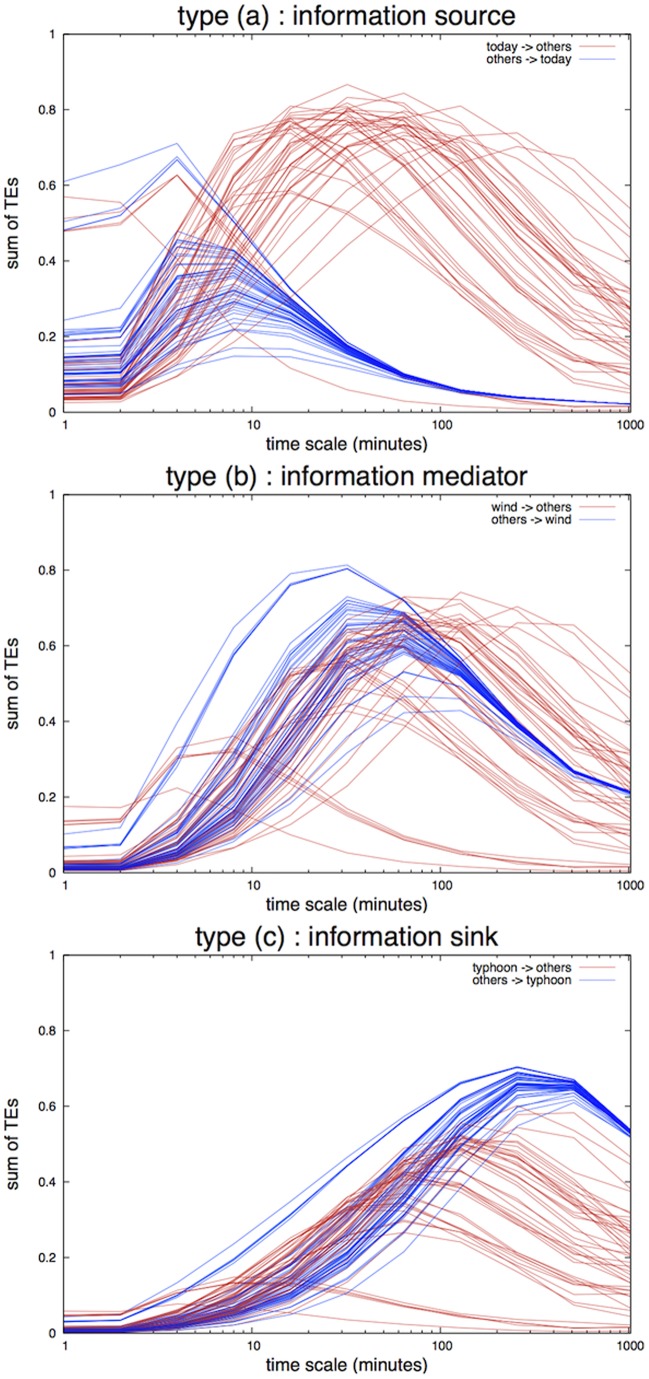
Integrated TE of a given keyword to/from the other 46 keywords by varying the time resolution 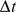 **from** 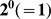 **minute to** 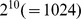 **minutes.** *Type a*) a keyword (e.g., “today” ranked 1st) acts as an information source, *type b*) a keyword (e.g., “wind” ranked 256th) acts as an information mediator, and *type c*) a keyword (e.g., “typhoon” ranked 551st) acts as an information sink.

We distinguished the results into three TE types as shown in Figure 6. The first one is *type a* whereby the TE from this keyword to other keywords is smaller than the TEs from other keywords to this keyword in the smaller 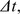 and the tendency is reversed in the larger 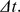 For example, the TE from other keywords to this keyword has the maximum TE value of around 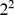 minutes and the TEs from this keyword to other ones have a maximum value of approximately 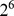 minutes (or approximately one hour). That is, if 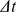 is larger than one hour, the direction of the TE flow is always from this keyword to other keywords. The second one is *type b*, whereby the TE from this keyword overlaps with the TEs from other keywords to this keyword. That is, there is no stationary TE flow direction between this keyword and other keywords. Finally, the third is *type c* whereby the TE from this keyword to other keywords is larger than the TEs from other keywords to this keyword in the smaller 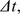 and the tendency is reversed in the larger 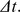


From this observation and judging from the direction of the TE flows, we identify *type a* as an information source to other keywords, *type b* as an information mediator, and *type c* as an information sink to other keywords. While many keywords act as *type b* for most of the time resolutions, interestingly, the higher-ranking keywords tend to play as *type a*, the information source, and the lower-ranking keywords tend to play as *type c*, the information sink. As evident from these figures in Figure 6, the effective 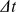 that maximizes the TE is different for each keyword. For the purpose of comparison, we used the time resolution 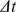 of one hour to compute the TE.

Finally, we investigated how each keyword plays a role as source, sink, or mediator for the 1,000 keywords. We computed the sum of all incoming flow (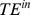) and all outgoing flow (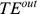) for each keyword. If 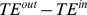 is greater than a given threshold, we say that the keyword plays the role of the information *source* for other keywords. Similarly, if 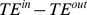 is greater than the threshold, we say it plays the role of the information *sink* to other keywords. This corresponds to *type a* and *type c*, respectively. We can further distinguish keywords that are neither source nor sink. If both 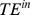 and 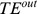 are smaller than the threshold, we refer to this keyword as a *weak mediator*. On the other hand, if both 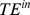 and 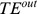 are greater than the threshold, we refer to this keyword as a *strong mediator*. These mediator nodes do not become either sink or source but act as “relay” nodes to send and receive information to other keywords.

**Figure 7:**
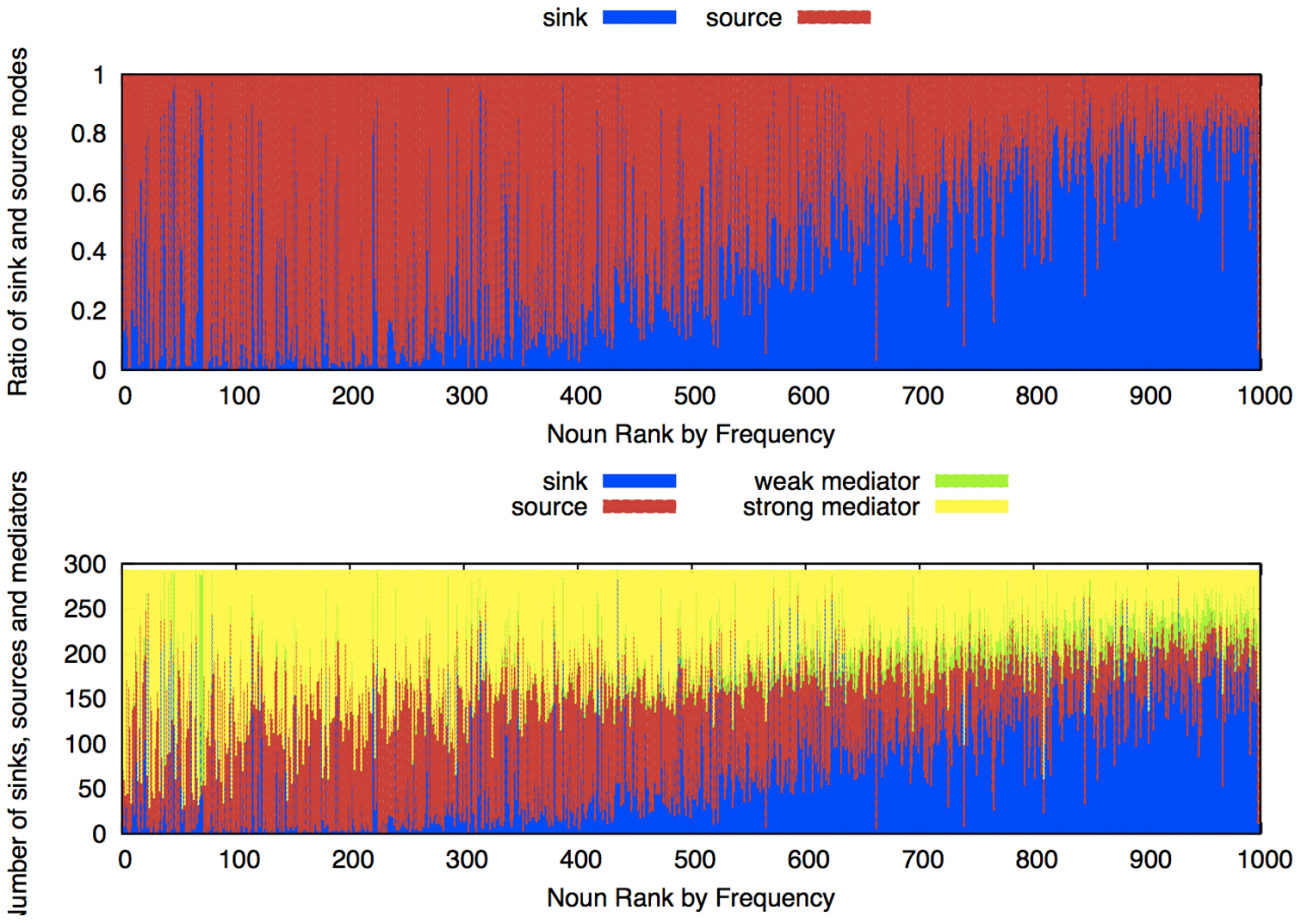
The role of each keyword. (Top) The ratio of keywords becoming sources and sinks shown as a function of keyword frequency over time. Red shows the source ratio and blue shows the sink ratio, as a function of keyword frequency over time. The frequent keywords tend to become source nodes and infrequent keywords tend to become sink nodes. (Bottom) Strong mediators are defined as having ample incoming and outgoing TE flow and weak mediators are defined as those with both weak incoming and outgoing TE flow. The number of strong and weak mediators is incorporated with the number of sinks and sources for each keyword.

The result of the computations for the 1,000 keywords is depicted in Figure 7. The TE was created from the 240-hour time window for over 302 days. We characterize each keyword by labeling it as source, sink, weak mediator, or strong mediator. We used the threshold however, of course, these labels, either source or sink, are not fixed but temporally changing as with the advent of time. If keywords are purely uncorrelated, the rate of becoming sink or source must be flat against the keyword rank order. Instead, Figure 7-(top) shows that frequent keywords have a higher tendency to become source nodes when compared to less-frequent keywords.

**Figure 8:**
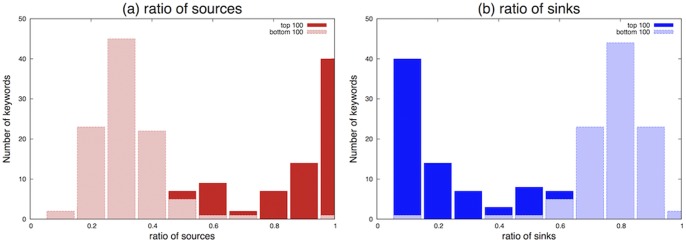
A histogram of the ratios for each keyword that becomes sources a) and sinks b) computed from the top 100 and bottom 100 keywords. The top 100 keywords have a higher ratio of becoming sources compared to the bottom 100 keywords, whereas the bottom 100 keywords have a higher ratio of becoming sinks compared to the top 100 keywords.

To investigate this characteristic further, the top 100 and bottom 100 keywords were investigated to determine their tendencies to become source or sink. Figure 8 shows that the top 100 keywords tend to become an information source, whereas the bottom 100 keywords have the opposite tendency; i.e., they tend to become an information sink. Table 1 shows the top 10 most-frequent source keywords and the top 10 most-frequent sink keywords out of 1,000 keywords. We see that pronouns such as “I” or “everybody,” and ordinary nouns that are used in everyday situations such as “lunch” or “thing,” become source nodes. More generic keywords, such as “game” or “play,” and some specific words, such as “JST (Japan Science and Technology Agency)” or “Nihonbashi” (a district in Tokyo), become sink nodes. Figure 7-(bottom) shows the actual number of source, sink, and mediator nodes for all the windows. As we see in the figure, frequent keywords tend to become source nodes from the perspective of the sink/source ratio, but they also tend to become relay nodes.

**Table 1:** The top 10 source and sink keywords from among 1000 keywords.

Rank	source keywords	Rank	sink keywords
767	lunch	779	RJTY
567	lunchtime	526	game
59	thing	38	mm
48	direction	574	where
9	I	619	map
63	long time	316	Nihonbashi
49	eye	625	play
76	everyone	437	average
7	now	226	JST
100	for	44	wind

We argue that these Twitter time series are not independent of each other. When a keyword is an information *source*, knowing the time series of the keyword tends to reduce the uncertainty of the other keywords. When a keyword is an information *sink*, knowing the time series of the other keywords can reduce the uncertainty of that keyword. In brief, our contention is that frequent keywords have a strong tendency to become source nodes and infrequent ones to become sink nodes. Since frequent keywords are less driven by the real world because they have fewer bursts, we conclude that frequent keywords are mainly activated by their inherent dynamics (e.g., weak correlation through timelines) and that they form the default mode of the Web. Frequent keywords as an information source means they can reduce uncertainty in the time series of infrequent keywords. The default mode can be an important Web mode, not only for supporting baseline activity but also for reducing uncertainty in information circulation on the Web, thereby regulating the consistency of information between the Web and the real world. This is consistent with the roles of frequent keywords of Twitter as discussed in relation to the TEs between Twitter and Google.

## Discussion

This paper explored how to define the Web's reactive and default modes by information transfer by computing TE to characterize the inherent structure of the Web dynamics. First, we defined whether a keyword is in default or reactive mode in terms of how burst events are caused internally or externally. There are reports on YouTube page views and Twitter hashtags, whereby internally and externally caused bursts are distinguished by certain criteria [17], [19]. Our analysis of the number of bursts in relation to keyword frequency revealed that while low-frequency keywords tend to burst more, keywords are more influenced by real-world events, when compared to high-frequency keywords.

From this observation, we defined that high-frequency keywords form the Web's default mode network and low-frequency keywords constitute the Web's reactive mode. When analyzing the information transfer between Google and Twitter, we found that information is mostly transferred from Twitter to Google and that this tendency is more apparent for high-frequency keywords than for low-frequency keywords. We also studied the information flow network formed among Twitter keywords by taking the keywords as nodes and flow direction as the edges of a network. We found that high-frequency keywords tend to become information sources and low-frequency keywords tend to become information sinks. These findings suggest that we can use high-frequency keywords (or default mode of the Web) to reduce uncertainty with the externally driven low-frequency keywords (or reactive mode of the Web). However, it is fair to assume that frequently searched keywords in Google are different from the frequent keywords found on Twitter. Thus, if we investigated the high-frequency keywords found in Google queries, the results may be different.

The concept of reactive and default modes originates from brain science [20]–[22]. A brain region responsible for a given task is identified by measuring the neural activity that is observably higher compared to the baseline activity. Raichle et al. [23] examined the baseline activity by analyzing the regions that become less active when a specific task is given. This successful approach uncovered some remarkable perspectives and characteristics of the default mode; based on Buckner's [22] and Raichle's [24] reviews, these are: i) the area associated with the default mode is found as the integration of various subsystems in the brain - the medial prefrontal cortex and posterior cingulate cortex subsystems seem to play central roles. ii) The neural activity of the aforementioned subsystems were observed as noisy fMRI signals at a low frequency of about 0.1 Hz or less, showing global synchronization. iii) The default mode is to do with spontaneous cognition e.g., day dreaming and internal thoughts such as future planning. iv) The activity of the default mode is anti-correlated with the other brain regions that are responsible for focusing attention on the external world; and v) the brain region associated with the default mode overlaps with those involved in the construction of episodic memory.

This notion of the default mode can be generalized for any living systems with or without brain systems. In the case of the Web system, it can be said that 1) frequent keywords constitute the default mode (mostly everyday keywords), 2) these frequent keywords display less frequent bursting behaviors and are an information source for other keywords, 3) the default mode may help reduce uncertainty in the entire Web system, and 4) the default mode comprises quasi-periodic time series. From this comparison with the default mode network in brain systems, and in particular with the possibility that high-frequency keywords may help to predict essentially unpredictable events, it becomes apparent the Web's default mode may have the same property as the default modes in the brain. Differentiating between these two modes, the reactive and the default, provides a useful perspective for understanding Web dynamics and predicting the future of bursting behavior in the time series of keyword frequencies in tweets in Twitter, as well as in the time series of search queries in Google. With respect to the examples of complex networks in general, we believe that the default mode is key for understanding autonomy in complex systems in general. Any autonomous system (e.g., robots) possesses primitive forms of the default mode with different time scales [25].

Determining the adequate time resolution in a time series is generally a difficult problem, particularly when we compute TE. In Twitter time series, tweets are basically created out of individual users' postings with no simple threshold or global knowledge of who is posting. With the apparent 24-hour periodicity that we see in keywords like “good night,” it is rather appropriate to use a one-hour resolution to analyze information transfer and correlation. On the other hand, the keyword “Christmas” has a one-year periodicity and keywords like “earthquake” have no characteristic time resolutions. However, when we examine the time resolutions of the order of a few minutes to a few hours, these time series are similar.

In the section entitled **Determining the Time Resolution** 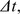 we varied the time resolution of each time series and computed the information transfer to ascertain how the TE changes as a function of the time resolution. The TE between Google and Twitter is relatively robust against the change of 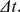 Moreover, with regard to TE among different Twitter keywords, the maximum value of outgoing TE is found around one hour for many keywords, but there are exceptions. Due to the limitation of data availability and computational power, we chose one day as the time unit for calculating TE between Twitter and Google, and one hour as the time unit for keywords within Twitter.

We may have to adopt different time resolutions for different keywords. Namely, some keywords provide more information within a smaller time resolution but some provide information in much longer time resolutions. As a result, the total uncertainty of Twitter is distributed over different time resolutions. Which time resolution is important for a keyword is determined by the dynamics of other keywords. For example, studies on nonlinear phase coupling systems show that faster phase oscillation generally entrains the slower one. Here, the situation becomes more complicated since the system is always perturbed by periodic or aperiodic open flow in the real-world. Moreover, the time scale hierarchy is self-organizing rather than given from the beginning. Google has an additional source of time scales on the Web such as automated crawling programs that change the search results that are running on the Web. The default and reactive modes that we found on the Web are the outcomes of such nested time architectures. The investigation of nested time scales may also be applicable to other complex adaptive systems.
